# The Impact of the COVID-19 Pandemic on Urgent Awake Tracheotomies in Adults

**DOI:** 10.7759/cureus.44015

**Published:** 2023-08-24

**Authors:** Ozlem Yagiz Agayarov, Ilker Burak Arslan, Murat Gumussoy, Gulay Guclu, Ibrahim Cukurova

**Affiliations:** 1 Department of Otolaryngology, Head and Neck Surgery, Tepecik Training and Research Hospital, Health Sciences University, Izmir, TUR

**Keywords:** upper airway obstruction, stay suture, head and neck malignancy, covid-19 pandemic, urgent awake tracheotomy

## Abstract

Objective

This study aims to investigate the impact of the COVID-19 pandemic on urgent awake tracheotomies (UATs) in adults in a reference center.

Methodology

During the pandemic (between March 2020 and May 2022) and before the pandemic (between January 2018 and March 2020), medical charts of patients who underwent UATs were reviewed. The analysis focused on demographic characteristics, indications, COVID-19 positivity status, surgical procedures, and complications.

Results

During the pandemic, 67 UATs (age 62.04 ± 11.9 years) were performed. Of the indications, 56 (83.5%) were malignancy. Before the pandemic, 22 UATs (age 63.86 ± 15.1 years) were performed, of which 14 UATs (63.6%) were due to malignancy. There was a significant increase in UATs and their indications in patients with head and neck malignancies (*P *< 0.05). Stay suture (65, 97%) and suture ligation of the thyroidal isthmus (61, 91%) were significantly performed during the pandemic (*P *< 0.05).

Conclusions

A significant increase in UAT was detected, especially in patients with advanced head and neck cancer. Pandemic conditions and the risk of viral transmission have led to more conservative UAT techniques.

## Introduction

Urgent awake tracheotomies (UATs) are indicated for acute upper airway obstruction when the patient cannot be intubated or cannot oxygenate [1]. The preferred procedure for establishing urgent airway access is open tracheotomy between the first two to three tracheal rings while the patient is awake [2]. After tracheotomy, ideally, patients should be followed up under intensive care conditions by experienced healthcare professionals.

Different techniques are employed to prevent tracheotomy complications, particularly hemorrhage and early tube displacement. We have performed hemostasis by electrocautery or suture when a thyroid isthmus incision is needed. Stay sutures can be placed through the tracheal wall or tracheal flap. These sutures can be used to identify the trachea and reinsert an inadvertently dislodged tracheostomy tube. These can be taped to the neck skin on either side to facilitate tube replacement and/or tacked to skin edges to create a semipermanent stoma [3].

The COVID-19 pandemic created overwhelming challenges and some major modifications in the healthcare system worldwide [4,5,6]. In our country, access to the health system decreased due to stay-at-home orders, patients’ fear of visiting hospitals, the closure of outpatient clinics, and the conversion of some levels 1 and 2 hospitals into pandemic hospitals. The COVID-19 outbreak has disrupted diagnosis, planning, initiation, and duration of treatment in most ENT diseases, particularly head and neck cancers [7,8].

The study aimed to investigate the challenges and diversity of UATs during the COVID-19 pandemic, focusing on patient characteristics and surgical experiences, and to compare data from both before and during the pandemic.

## Materials and methods

The study was approved by the Local Ethics Committee of Tepecik Training and Research Hospital (Ethics Committee decision no. 2022/08-17).

All patients who underwent UATs between March 11, 2020 (our country announced its first case of coronavirus) and May 16, 2022 (coronavirus containment measures lifted) were analyzed retrospectively and compared with a similar time interval (26 months) before the pandemic (January 2018 to March 2020). The otorhinolaryngologist and the anesthesiologist decided to conduct UATs after evaluating the airway with rigid or fiberoptic endoscopes (Video 1). All UATs were performed by using the open technique under local anesthesia with monitoring and oxygen support. Thyroid isthmus divided with electrocautery and sutured continuously to avoid bleeding due to irritation of the tracheotomy tube. We performed U-shaped, superior-based tracheal flaps for patients who required temporary airway passage with unknown etiology and who were candidates for laryngectomy (Video 2). During the pandemic, all UATs were performed with the protective equipment recommended by the World Health Organization. Before the pandemic, surgical masks and protective glasses were used only.

**Video 1:** Endoscopic examination of a patient with severe dyspnea due to a laryngeal mass. Endoscopic examination image of a 57-year-old male patient who presented with severe dyspnea due to a laryngeal mass.

**Video 2:** Performing U-shaped, superior-based tracheal flap.

Demographic characteristics, indications, COVID-19 positivity, the ratio of emergent to the total number of tracheotomies, the performed tracheotomy technique, and complications were analyzed. Information regarding patients who underwent tracheotomy by our department and those who were subsequently transferred to other hospitals was acquired from the national health database. COVID-19 polymerase chain reaction (PCR) tests were conducted on all patients, encompassing both nasopharyngeal and oropharyngeal samples, before undergoing UAT. Data collected at a similar time interval (26 months) during the pandemic and before the pandemic were compared.

Statistical analysis

The data obtained in this study were analyzed with the IBM SPSS Statistics Version 21 (IBM Corp., Armonk, NY, USA) package program. Descriptive analyses were used to evaluate the parameters. Chi-square and t-test analyses were utilized to explore relationships between groups. A significant relationship was noted when *P *< 0.05, while a lack of significant relationship was indicated when *P *> 0.05.

## Results

In the 26 months before the pandemic, 22 UATs were performed (Table 1). The mean age of the patients was 63.86 ± 15.1 (7 females, 15 males). Of the 22 UATs performed, 14 (63.6%) were attributed to malignancy, 3 (13.6%) to infectious causes, 1 (4.5%) to trauma, 1 (4.5%) to bilateral VC paralysis, 1 (4.5%) to subglottic stenosis, and 2 (9%) to intraoral bleeding. Of the 22 tracheotomies, 4 (18.18%) were performed during the night shift. Of the 247 tracheotomies performed in the same period, 22 (8.9%) constituted emergency tracheotomies (Table 2).

**Table 1:** Demographic characteristics of patients, preferred modifications for tracheotomies, and complications of tracheotomies. *P* < 0.05 is accepted as a significant difference. SD, standard deviation; UAT, urgent awake tracheotomy

Demographics	Before pandemic (*n* = 22)	During pandemic (*n = *67)	P
Age (years) (mean ± SD)	63.86 ± 15.1	62.04 ± 11.9	0.67
Female/Male, *n* (%)	7/15 (46.6)	12/55 (21.8)	0.167
Tracheotomy: urgent/elective, *n* (%)	22/247 (8.9)	67/266 (25.1)	0.008
Patient from the emergency department/otorhinolaryngology outpatient clinic, *n* (%)	6/16 (37.5)	23/44 (52.2)	0.54
Night shift/daytime, *n* (%)	4/18 (22.2)	18/49 (36.7)	0.412
Thyroid isthmus suture ligation, *n* (%)	9 (40.9)	61 (91)	0.000
Stay suture, *n* (%)	8 (36.3)	65 (97)	0.000
Dislocated tube, *n* (%)	1 (4.5)	2 (2.98)	0.724
Pulmonary edema, *n* (%)	2 (9)	3 (4.5)	0.414
Early postoperative bleeding, *n* (%)	1 (4.5)	1 (1.5)	0.401
Patient transferred for tracheotomy, *n* (%)	4 (18.1)	34 (50.7)	0.007
Patient transferred after UAT, *n* (%)	1 (4.5)	21 (31.3)	0.015

**Table 2:** Head and neck malignancy and distribution of laryngeal cancer and treatments. *P* < 0.05 is accepted as a significant difference. UAT, urgent awake tracheotomy; CT, chemotherapy; RT, radiotherapy

	Before the pandemic (*n = *22)	During the pandemic (*n = *67)	P
Head and neck malignancy, *n* (%)	14 (63.6)	56 (83.5)	0.047
Laryngeal cancer, *n* (%)	7 (31.8)	37 (55.2)	0.056
Laryngectomy after UAT, *n* (%)	4 (18.1)	13 (19.4)	0.272
CT-RT after UAT for laryngeal cancer, *n* (%)	2 (9)	13 (19.4)	0.736
Exitus before starting treatment for laryngeal cancer, *n* (%)	1 (4.5)	11 (16.4)	0.4

During the 26 months of the pandemic, a total of 67 UATs were conducted. The mean age of the patients was 62.04 ± 11.9 (12 females, 55 males). Of the 67 UATs performed, 56 (83.5%) were attributed to malignancy, 3 (4.4%) to infectious causes, 2 (2.9%) to bilateral vocal cord (VC) paralysis, 2 (2.9%) to benign lesion compressing on the larynx, 2 (2.9%) to subglottic stenosis, 1 (1.4%) to trauma, and 1 (1.4%) to difficult intubation. Of 56 head and neck cancers, 37 (66%) were attributed to laryngeal cancer, 6 (10.7%) were hypopharyngeal cancer, 3 (5.35%) to esophageal cancer, 2 (3.57%) to oral cavity cancer, 2 (3.57%) to oropharynx cancer, 2 (3.57%) to neck metastasis of nasopharyngeal cancer, 1 (1.78%) was maxillary sinus cancer, and 3 (5.35%) to head and neck carcinoma of unknown primary. Of the 67 patients who underwent UATs, 23 (34.3%) were referred to the emergency department. Of the 67 tracheotomies, 18 (26.8%) were performed during the night shift. Of 37 laryngeal cancers, 28 (75.6%) were transglottic, 5 (13.5%) were supraglottic, and 4 (10.8%) were glottic (two recurrences after radiotherapy) cancers. Laryngeal biopsy was performed at the same time in 26 (38.8%) patients. Thirteen (19.4%) patients with biopsy-confirmed squamous cell carcinoma underwent surgery. A total of 12 (17.9%) total laryngectomies and 1 (1.5%) supraglottic laryngectomy were performed along with bilateral neck dissection. Eight (11.9%) patients were deemed inoperable, and 5 (7.5%) patients who declined surgery were referred to the oncology clinic. In this period, of 266 tracheotomy cases, 67 (39.7%) were UATs. It was known that 3 (4.4%) patients were COVID-19-positive and 41 (61.19%) patients were COVID-19-negative before tracheotomy. A total of 23 (34.3%) UATs were performed without COVID-19 test ended due to severe dyspnea. These patients were isolated after tracheotomy, and 2 of these patient’s tests were reported as positive. Of 67 patients, a stay suture was used in 65 (97%).

During the pandemic, emergency tracheotomies resulted in major postoperative complications, including pulmonary edema (3, 4.5%), tube dislodgement (2, 2.9%), and early postoperative bleeding (1, 1.5%), all of which were successfully managed.

## Discussion

Tracheotomy, an aerosol-generating procedure that increases healthcare workers' exposure to COVID-19 infection, became a challenging procedure during the pandemic. We aimed to present the experience of our referral center and compare the indications, outcomes, and complications of UATs.

The COVID-19 pandemic caused delays in the diagnosis and treatment of many diseases such as head and neck cancers [9]. The reasons could be the situation of primary care, blockage of hospital visits, less patient consultation, etc. Also, many hospitals where head and neck cancer patients were previously treated had turned into pandemic hospitals, and ENT specialists dealt with COVID-positive patients instead of ENT patients. The admission of these patients was often delayed until the advanced stages, sometimes even when acute airway obstruction was present. We observed a notable rise in patients undergoing UATs during the pandemic, especially in cases of delayed admission for advanced head and neck cancer.

During the pandemic, it was not always possible for patients to be followed up by their surgeons or experienced post-operative care teams. During the pandemic, Level 2 and Level 3 intensive care units were transformed into pandemic intensive care units. Experienced healthcare workers were reassigned to other health institutions that were repurposed as pandemic hospitals, and some patients had to be transferred to other healthcare facilities due to the demand for isolated beds and the limited capacity of intensive care units. We experienced that stay sutures facilitated the reinsertion of the tube even in the absence of the assistance of ENT doctors or experienced healthcare professionals. These reasons may explain the statistically significant use of the stay suture.

Although some authors recommend Heffner’s subthyroidal tracheotomy with retraction and preservation of the thyroid isthmus in emergency tracheotomy, the majority of surgeons use suture ligate or divide the thyroid isthmus with cauterization accompanied by meticulous hemostasis to prevent hemorrhage [10]. Minimizing the risks of postoperative bleeding and duration of surgery became more important during the pandemic. Suture ligation of thyroid isthmus and avoiding low tracheotomy should decrease postoperative bleeding while prolonging the duration of the operation. According to this study, thyroid isthmus suture ligation was used significantly during the pandemic. Preference of suture ligation may include decreasing aerosol exposure by limiting electrocautery and hemorrhage [11,12].

Tracheotomy before laryngectomy complicates intraoperative macroscopic and postoperative microscopic evaluation of surgical margins and increases stomal recurrence risk in advanced laryngeal cancer, especially in subglottic extension [13]. It is difficult to evaluate the larynx and stage the tumor in patients presenting with acute airway obstruction with endoscopic examination alone. Superiorly based tracheal flap (SBTF), which can easily return to its original position after decannulation, is less harmful to the patient than an irreversible tissue excision such as a tracheal window. If early decannulation is indicated, returning the tracheal flap to the original position may restrict tracheal secretions and aerosol generation. In addition, most of our patients had transglottic laryngeal cancer. SBFT facilitates the formation of stomas and provides precise measurement of surgical margins. The preference for SBFT could be attributed to the time constraints for staging and the potential risk of aerosol exposure following early decannulation.

No instances of COVID-19 positivity were identified among any members of the operating team, which comprised a total of 6 surgeons, 11 resident surgeons, and 14 nurses, after completion of UATs on patients who were either COVID-19-positive or suspected to be positive. Considering the anxiety, choking sensation, coughing, and convulsive effort of the UAT patient, and associated aerosol transmission, standard COVID-19 prevention methods and our emergency tracheotomy approach can be considered adequate and safe.

George et al. highlighted the duration of the operation to minimize the exposure to aerosolized secretions and avoid low tracheotomy to facilitate a future laryngectomy. They used vertical skin incision and excised a piece of tracheal cartilage from the second or third ring [14]. Khanna et al. presented a total of 115 patients who underwent 117 emergency tracheotomies during a pandemic. Pre-tracheotomy COVID testing reports were available for only 11 patients, and 9 of them tested positive on post-tracheotomy COVID testing. The pyriform sinus was the most prevalent tumor site (45.2%), and 45% of patients presented with stage IVA [15].

This study has its limitations. First, it was a single-center, retrospective study. Second, data include a small population of the country. Third, it is very difficult to compare surgical preferences and complications and make clear recommendations due to the variability of patients' clinics and urgency.

## Conclusions

The COVID-19 pandemic caused delays in the diagnosis and treatment of many diseases, including head and neck cancers. A significant increase in emergency local tracheotomies, particularly for advanced head and neck malignancy was a result of disruptions in healthcare. The pandemic has had ramifications for surgical practices, postoperative care, and the protection of both healthcare workers and patients. Consequently, SBTF, tracheal stay sutures, and suture ligation of thyroid gland isthmus are significantly preferred by surgeons during the pandemic.
